# Neuroimaging as a Tool for Early Dementia Detection: Quality Improvement in Clinical Practice

**DOI:** 10.1192/bjo.2026.11254

**Published:** 2026-06-30

**Authors:** Ahmet Mete Demir, Jannah Khattak, Bushra Azam

**Affiliations:** 1 Derbyshire Healthcare NHS Foundation Trust, Derby, United Kingdom; 2 University of Buckingham, Buckingham, United Kingdom

## Abstract

**Aims:**

To review CT and MRI head scan reports of patients aged 65 years and over referred to the Mental Health Liaison Team, focusing on whether dementia-relevant features were consistently reported.

To examine the presence and reporting of cerebral atrophy, vascular changes and infarcts, and to compare acute hospital discharge diagnoses with final community psychiatric diagnoses in order to identify gaps in early recognition of dementia.

**Methods:**

This quality improvement project reviewed a randomly selected sample of 14 patients aged 65 years and over referred to the North Mental Health Liaison Team at Chesterfield Royal Hospital between 1 November 2025 and 31 January 2026. CT and MRI head scan reports requested during the acute admission were reviewed.

All data were anonymised in line with information governance requirements. Imaging reports were assessed for documentation of acute pathology, cerebral atrophy, vascular change and established infarcts. Acute hospital discharge diagnoses were compared with subsequent diagnoses made by community mental health services. Data were collected using a structured Microsoft Forms tool and analysed descriptively using Microsoft Excel.

**Results:**

Confusion was the primary indication for neuroimaging in approximately 90% of cases. Over 90% of scans reported no acute intracranial pathology. Cerebral atrophy, either global or focal, was documented in over 80% of scans, whilst vascular changes were reported in approximately 90%. Established infarcts were less consistently described, with variable emphasis on their relevance to cognition. At the point of acute hospital discharge, around 40% of patients were given a functional psychiatric diagnosis. Following community follow-up, approximately 40% of patients were ultimately diagnosed with dementia, indicating a significant shift in diagnostic formulation over time.

**Conclusion:**

Older adults presenting acutely with confusion frequently demonstrate chronic neuroimaging changes relevant to dementia, even when acute pathology is excluded. The proportion of patients later diagnosed with dementia suggests that opportunities for earlier recognition exist during the acute admission. More consistent and structured reporting of dementia-relevant imaging features, alongside closer collaboration between radiology and liaison psychiatry, may support earlier referral to memory services, improve continuity of care and reduce unnecessary repeat investigations.

